# Finding potential targets in cell-based immunotherapy for handling the challenges of acute myeloid leukemia

**DOI:** 10.3389/fimmu.2024.1460437

**Published:** 2024-09-30

**Authors:** Amir Hossein Kheirkhah, Sina Habibi, Mohammad Hasan Yousefi, Sara Mehri, Bin Ma, Mahshid Saleh, Maria Kavianpour

**Affiliations:** ^1^ Department of Tissue Engineering and Applied Cell Sciences, School of Medicine, Qom University of Medical Sciences, Qom, Iran; ^2^ Department of Hematology and Blood Banking, Faculty of Allied Medicine, Iran University of Medical Sciences, Tehran, Iran; ^3^ Department of Biotechnology, School of Paramedical Sciences, Qazvin University of Medical Sciences, Qazvin, Iran; ^4^ School of Biomedical Engineering, Med-X Research Institute, Shanghai Jiao Tong University, Shanghai, China; ^5^ Clinical Stem Cell Research Center, Renji Hospital, Shanghai Jiao Tong University School of Medicine, Shanghai, China; ^6^ Wisconsin National Primate Research Center, University of Wisconsin Graduate School, Madison, WI, United States; ^7^ Cellular and Molecular Research Center, Qom University of Medical Sciences, Qom, Iran

**Keywords:** acute myeloid leukemia, cell therapy, immunotherapy, CAR-T cell, TCR-T cell, natural killer cell

## Abstract

Acute myeloid leukemia (AML) is a hostile hematological malignancy under great danger of relapse and poor long-term survival rates, despite recent therapeutic advancements. To deal with this unfulfilled clinical necessity, innovative cell-based immunotherapies have surfaced as promising approaches to improve anti-tumor immunity and enhance patient outcomes. In this comprehensive review, we provide a detailed examination of the latest developments in cell-based immunotherapies for AML, including chimeric antigen receptor (CAR) T-cell therapy, T-cell receptor (TCR)-engineered T-cell therapy, and natural killer (NK) cell-based therapies. We critically evaluate the unique mechanisms of action, current challenges, and evolving strategies to improve the efficacy and safety of these modalities. The review emphasizes how promising these cutting-edge immune-based strategies are in overcoming the inherent complexities and heterogeneity of AML. We discuss the identification of optimal target antigens, the importance of mitigating on-target/off-tumor toxicity, and the need to enhance the persistence and functionality of engineered immune effector cells. All things considered, this review offers a thorough overview of the rapidly evolving field of cell-based immunotherapy for AML, underscoring the significant progress made and the ongoing efforts to translate these innovative approaches into more effective and durable treatments for this devastating disease.

## 1 Introduction

Acute Myeloid Leukemia (AML) is a very assertive and genetically diverse hematologic malignancy that can be life-threatening if not treated (1). Flow cytometry is the primary diagnostic tool for assessing surface antigens on leukemia cells, as simple morphology is insufficient for lineage determination (2). Special histochemical stains are also necessary, while peripheral blood can aid in diagnosis, a bone marrow biopsy is essential for evaluating morphology, cell surface markers, and for cytogenetic and molecular analysis. For a diagnosis, peripheral blood or bone marrow must have a blast count of 20% or above, except in specific cases with certain chromosomal abnormalities (e.g., t(15;17), t(8;21), inv(16), or t(16;16)) (3). The prognosis for AML is unfavorable, with cure rates of 5-15% in patients over 60 years old and 35-40% in those under 60 years old (4). AML genetic changes make cells grow faster, stop leukemic cells from maturing, and slow down programmed cell death (5). This causes cancerous cells to replace healthy erythroid, myeloid, and megakaryocytic progenitors (6). Clinically, AML manifests with low blood cell counts, including anemia, infections, bleeding, and bruising, alongside general symptoms, metabolic irregularities, and various complications (7). Cytogenetic and molecular events significantly impact AML subgroup classification and clinical management (8). AML is classified into two subtypes under the 5th edition of the World Health Organization’s (WHO) classification of hematolymphoid tumors: AML defined by differentiation and AML with defining genetic abnormalities (9). Genetic abnormalities remain essential diagnostic criteria.

Over the past 35 years, several studies have established a therapeutic induction protocol, now deemed the gold standard for patients not participating in clinical trials (10). This “3 + 7” protocol, which marries anthracycline and cytarabine, has emerged as the most potent intervention for AML. The primary objective of this therapeutic approach is eradicating leukemic cells from both the circulatory system and the bone marrow (BM) (11, 12). In certain instances of AML, however, high-dose cytarabine or hematopoietic stem cell transplantation (HSCT) may be effective ways to treat the disease (13). While many patients respond well to first-line therapy and find symptomatic relief, merely a little portion achieve long-term survival due to chemotherapy-resistant relapses (14). Additionally, challenges like drug resistance, limited therapy options for specific patient groups, and the urgent need for more effective targeted therapies present significant obstacles in AML treatment (15). Furthermore, it is possible to have difficulties obtaining and financing medical care and effectively handling the adverse effects of treatment. These deficiencies underscore the urgent need to address these gaps to improve outcomes for individuals battling AML (16). Despite advancements in the treatment of AML, particularly in higher-risk populations, progress in prolonging survival remains slow. Complete remission (CR) rates have increased since targeted treatments were introduced in conjunction with chemotherapy; however, relapse rates have not significantly changed, with over 60% of patients experiencing relapse, leading to a median disease-free survival (DFS) of less than one year (17). This emphasizes the urgent must recognize more effective therapeutic targets and enhance the efficacy of targeted therapies. While consolidation therapy has demonstrated benefits in improving overall survival (OS), its efficacy varies among patients, and maintenance therapy’s place in AML is still debatable (18). Few maintenance therapies, aside from arsenic trioxide and retinoic acid in acute promyelocytic leukemia, have shown adequate effectiveness to be adopted as standard treatment (17). Current induction therapies often fail to completely eliminate leukemic clones, necessitating additional post-remission approaches, especially for patients with adverse biological features (19). Allogeneic hematopoietic cell transplantation (allo-HSCT) is the most effective post-remission therapy but is associated with significant toxicities that may outweigh its benefits in certain patient groups. Furthermore, the detection of minimal residual disease (MRD) is crucial for identifying patients more likely to relapse and may inform maintenance treatment decisions (20). Historical maintenance strategies have often utilized drugs comparable to those employed in consolidation and induction, limiting their effectiveness. However, emerging clinical trials are exploring novel targets and maintenance approaches that incorporate targeted therapies based on specific mutational statuses, potentially offering improved safety and efficacy (21). Addressing these challenges through the identification of better targets and the refinement of targeted therapies is vital for advancing treatment outcomes in AML.

The evolving landscape of cancer treatment is shifting towards targeted therapies that enhance the host’s immune system to mount effective antitumor responses against malignancies (22). Immunotherapy, in particular, has experienced remarkable progress, solidifying its position as a cornerstone of cancer management alongside established methods such as surgery, chemotherapy, and radiotherapy (23). Immunotherapy has become an effective treatment approach against various human cancers, leveraging the immune system’s power to eliminate malignant cells (24). This method is broadly classified into two categories: active and passive immunotherapy. Active immunotherapy primarily involves utilizing dendritic cell vaccines, which stimulate the ability of the immune system to identify and target cancer cells (25). Conversely, passive immunotherapy encircles a range of strategies that directly enhance or modify immune cells to combat cancer (26). These include chimeric antigen receptor T (CAR-T) cell therapy, natural Killer (NK) cell therapy, and T cell receptor-engineered T (TCR-T) cell therapy. Moreover, passive immunotherapy extends to utilizing checkpoint inhibitors, which disengage the immune system’s brakes, enabling it to combat cancer more effectively. Passive immunotherapy also uses oncolytic viruses, which only infect and kill cancer cells, and monoclonal antibodies, which attack specific proteins in cancer cells (27, 28). By harnessing these immunotherapeutic strategies, there is potential for better results for AML patients, particularly in those with relapsed or refractory disease. This study aims to examine the most current developments in AML immune cell treatments, evaluate the current obstacles in this area, and introduce developing approaches that could improve treatment efficacy.

## 2 Immune cell therapy targeting specific antigens for AML

HSCT has proven highly effective in curing AML, yet it carries risks of transplant-related complications and fatalities. As a result, many patients succumb to disease progression or recurrence as a result of unfavorable side effects or treatment resistance (29). Unfortunately, not all patients qualify for HSCT, and relapse following transplantation continues to be the main reason for treatment failure. Consequently, exploring innovative AML treatments is vital (30). Harnessing the immune system to eradicate leukemic cells shows a great potential therapeutic approach, with successful implementations in HSCT and non-HSCT settings. Immunotherapies that use immune cells, like CAR-T cells, TCR-T cells, and CAR-NK cell therapy, have proven to be very efficient at treating AML invulnerable to chemotherapy (31). This section will explore various cell-based immunotherapies, examining their unique mechanisms and potential applications in AML treatment.

### 2.1 CAR-T cell therapy

CAR-T cells have revolutionized the therapeutic environment for individuals with lymphoma, acute lymphoblastic leukemia (ALL), and multiple myeloma (MM), demonstrating remarkable clinical success and significantly improving patient survival rates. The US Food and Drug Administration (FDA) has authorized CAR-T cell products for these signs, highlighting their therapeutic potential (32). On the other hand, CAR-T cells need to be used right away to treat AML, where treatment resistance (10–40%) and relapse are still big problems, especially for people who can’t get allogeneic HSCT (33). Since the arrival of cutting-edge technology, CAR-T cell engineering is making significant strides in AML treatment. The focus on enhancing specificity, reducing toxicity, and improving efficacy is set to transform our procedure to AML therapy (34). One of the recent advances in CAR-T cell therapy for AML involves targeting specific antigens like CD33, CD123, FLT3, and CLL-1, among others. Clinical trials are currently in progress to assess the security and performance of CAR-T cells in relation to these targets (35).

Several challenges it has to be addressed to treat AML using CAR-T cells effectively. These include the absence of an appropriate antigen uniformly expressed on leukemic cells, the intricate microenvironment of AML, and the need for suitable cell sources (36). Both preclinical and clinical studies have zeroed in on several antigens, such as CD33, CD123, CLL-1, and CD13, among others, as potential targets (37). Recent research has introduced the development of fourth-generation CAR-T cells, engineered with an immune modulator, which exhibit enhanced effectiveness and durability, potentially overcoming the tumor microenvironment (38). Exploring CAR-T cell therapy in AML treatment has unveiled new targets, notably leukocyte immunoglobulin-like receptor B4 (LILRB4) and Sialic acid-binding Ig-like lectin 6 (Siglec-6). These targets demonstrate a high degree of selectivity and low toxicity compared to normal hematopoietic cells (39). In refractory AML patients, compound CAR (cCAR) T-cells targeting multiple AML antigens, like CLL1 and CD33, have exhibited robust anti-tumor activity. This approach holds the potential to eliminate leukemia progenitor cells, paving the way for complete remission (40). Bi-specific CAR-T cells, intended to reach both CD13 and T cell immunoglobulin and mucin-containing-3 (TIM3), have shown assurance in potentially eradicating AML while minimizing toxic damage to human BM stem cells (41). A study by Sauer et al. underscores the effectiveness of CD70-specific CAR-T cells against AML, preserving normal HSCs and striking a harmony between safety and effectiveness (42).

Over 190 clinical trials are underway to make CAR-T cell biology more efficient and identify new targets, suggesting a promising future for CAR-T cell therapy in treating AML (34). A number of strategies are being investigated to improve the efficacy of CAR-T cell therapy, such as employing nanobodies to target specific antigens like CD13 and TIM3 (43). Studies conducted *in vivo* and *in vitro* have demonstrated that CAR-T cells target surface protein., including CD7, CD13, CD25, CD32, CD33, CD38, CD44, CD45RA, CD47, CD70, CD96, CD123, CLL-1, NKG2D ligand, Lewis Y antigen, Folate receptor β, FLT-3, CLEC12A, and TIM3, effectively eliminate AML cells (44) (**Table 1**).

**Table 1:** Clinical studies performed with targeting antigens as immunotherapy in AML treatment.

Type of cell-based Immunotherapy	Targeted Antigen	Function	AML Type	Clinical Phase	Outcome/limitation	References
CAR-T cell	CD7	Lymphocyte development	R/R AML	Phase I/II	• Non-myeloablative treatment of CD7^+^ AML	(45, 46) [NCT04762485] [NCT04033302]
	CD19	Transmembrane proteins that Facilitate survival of B-cell development.	t (8;21) in AML	Phase II/III	• Minimal residual disease • Leukemia relapse • Biased allocation of patients • incompleteness of immunophenotypic records	(47, 48) [NCT04257175]
	CD25	Type I transmembrane protein	R/R AML	Terminated	• No active clinical trial	(49–51) [NCT02588092]
	CD33	Modulate immune cellular processes (such as phagocytosis, cytokine release, and apoptosis.	R/R AML	Phase I/II	• ADCC through dual targeting • Survival • Safety Profile • Complete remission • Hematologic and Hepatic toxicity • Limited Long-term Efficacy	(52–56) [NCT05445765] [NCT06326021] [NCT06420063]
	CD34	Regulates cell differentiation, adhesion, trafficking, and proliferation.	–	–	• No active clinical trial	(57–59)
	CD38	Regulates calcium levels, NAD+ homeostasis, & Cyclic ADP-ribose hydrolase.	R/R AML	Phase I/II	• Enhanced cytotoxicity. • Got rid of CD38-positive blasts without harming lymphocytes or monocytes inadvertently.	(60–62) [NCT04351022]
	CD44v6	Displays many functions in healthy and diseased tissues by binding to hyaluronan, selectins, & osteopontin.		Phase I/II	• Monocytopenia • Dose limiting toxicity. • Inhibited proliferation • Induced differentiation and apoptosis • Eradicated AML LSC in PDX assays by affecting LSC trafficking to the BM niche.	(63–67)
	CD45RA	A unique marker for subpopulations of leukemia stem cells.	AML	Observational	• Anti-LSC/Effective treatment	(68) [NCT06297551]
	CD47 (IAP)	binds to the SIRPα protein’s N-terminus on immune cells to inhibit phagocytosis and provide a “do not eat” signal.	AML	Phase I	• Induced macrophage-mediated LSC killing. • Prevent the development of leukemia *in vivo*. • Improved phagocytosis • Improving the leukemic engraftment of AML cells in mice with NOD/SCID. • Eliminated AML LSC • • Resulting in long-term disease-free survival in PDX assays. • Effective treatment • Drug resistance	(69–75)
	CD56	Identification of two main NK-cell subsets: CD56^bright^ and CD56^dim^	R/R AML	Terminated	• No active clinical trial	(76)
	CD70	Upon activation, the TNF receptor ligand is transiently up-regulated on immune cells.	Newly Diagnosed AML	Phase I	• Reducing LSCs • Gene signatures that are trigged in relation to apoptosis and myeloid differentiation.	(77, 78) [NCT04662294] [NCT04227847]
	CD117 (c-kit)	Cell signal transduction is facilitated by the type III receptor tyrosine kinase.	R/R AML	Phase II	• Eradicated disease	(79) [NCT00707408]
	CD123	IL-3 receptor α-chain	R/R AML	Phase I/II	• Reducing off-tumor toxicities • Myelosuppression • Myelotoxicity • Toxicity related to targeting blood vessels.	(56, 80–83) [NCT03585517] [NCT03114670] [NCT02159495]
	CD174 (Lewis-Y, LeY)	o A carbohydrate antigen o Normal function in embryogenesis, tissue differentiation, tumor metastasis, and inflammation. o Overexpressed in hematological malignancies.	high-risk AML	Phase I	• The viability and security of CAR-T cell treatment • Durable *in vivo* persistence • Transient cytogenetic remission • Transient reduction of blasts • Stable disease	(84–86) [NCT01716364]
	CD276 (B7-H3)	Overexpressed in a sizable portion of AML patients’ leukemic blasts.	R/R AML	Unknown statue	• Stimulated the expansion and killing of T cells. • Stimulated the signal receptor of T cells. • Safety profile in preclinical models • Effective antigen-dependent cytotoxicity in AML xenograft and *in vitro* models	(87) [NCT04692948]
	CLL1 (CLEC12A)	Alters the state in which cells are activated during inflammatory processes.	Newly diagnosed & R/R AML	Phase I	• Reduced Dose Limiting Toxicities (DLTs) • Anti-leukemic activity • Extended survival • Effective and safe therapy • long-term prognosis of R/R AML	(88–90) [NCT03066648]
	FLT3 (CD135)	A class III cytokine receptor expressed on the surface of malignant blasts.	R/R AML	Phase I/II	• In xenograft models, potential suppression of leukemia proliferation • Eliminated primary AML blasts. • Using transplantation to rebuild the patients’ hematopoietic compartment High clinical value in the treatment of AML	(91–96) [NCT05023707] [NCT05017883]
	IL1RAP (IL1R3)	Indispensable for transmission of IL-1 signaling	AML	Not Applicable	• Anti-leukemic effects in xenograft models • Toxicity problems	(97) [NCT04169022]
	ILT3 (LILRB4)	An antidote to T cell growth and activation	AML M4/M5	Early Phase I	• Reduced tumor burden in an *in vivo* xenograft model without discernible negative effects on normal hematopoiesis.	(98) [NCT04803929]
	MUC1	Interact with receptor tyrosine kinases at the cell membrane and localize to the nucleus.	R/R AML	Phase I/II	• Effective depletion of AML cells *in vivo* without affecting normal hematopoiesis • Limited number of patient samples	(99)
	NKG2D-ligands	Bind directly to a wide range of ligand molecules that are expressed on the surface of cancerous cells.		Phase 1	• Improved CAR T-cell persistence • clinical responses	(100) [NCT02203825]
	Siglec-6	Connected to immune cells’ inhibitory signaling	Primary, secondary AML	Phase I/II	• Quick and effective removal of Siglec-6+ AML blasts	(101)
	WT1	A zinc-finger transcription factor is essential for the development and maturation of cells.	R/R AML	Phase I	• Significantly enhancing the survival of mice with AML • Limited to patients with HLA-A*02:01 • Restricting broader application.	(102–104)
**TCR-T cell**	MiHA HA-1H	Polymorphic peptides presented by HLA molecules.	high-risk AML	Phase I	• Decreased overall feasibility and efficacy. • Lack of TCR-T-cell expansion • Recurrent • Refractory	(105–107) [NCT03326921]
	PRAME	Inhibits cell differentiation, growth arrest, & apoptosis	R/R AML	Terminated	• No active clinical trial!	(103, 108, 109)
	WT1	Zinc-finger transcription factors are important for cell development and differentiation.	R/R AML	Phase I/II	• Immune reactivity • Immune escape • On-target toxicity • Reduced the risk of leukemic relapse. • Excellent safety record with no discernible on-target or off-tumor effects No severe adverse events. • Improved feasibility of clinical management of the protocol-specified population • Myelosuppression • Transient decreases of leukemic cells in bone marrow • Difficulties in the recruitment of patients.	(110–114) [NCT01621724] [NCT02550535]
**CAR-NK-cell**	CD33/CLL-1	Modulate immune cellular processes (such as phagocytosis, cytokine release, & apoptosis.	R/R AML	Phase I/II	• Several clinical trials are underway with so far promising results.	(89, 115, 116) [NCT02944162] [NCT05665075] [NCT05215015] [NCT05601466]
	CD112	Immune checkpoint inhibitor		Phase I	• Effective novel immunotherapy for AML.	(117, 118)
	CD123 (IL3Rα)	Regulates the proliferation, survival, and differentiation of hematopoietic cells.	R/R AML	Phase I	• Inhibited leukemogenicity in PDX assays. • Promising anti-leukemic activity • No CRS or neurologic toxicity	(82, 119–122) [NCT02159495] [NCT04230265]
	CAR-70/IL15	Upon activation, immune cells exhibit a temporary upregulation of the TNF receptor ligand.	R/R AML	Phase I/II	• No complete clinical trial	[NCT05092451]
	NKG2D ligand (NKG2DL)	Attach to a broad range of ligand molecules that are expressed on the surface of cancerous cells.	R/R AML	Phase I	• Small number in PB • Low ADCC activity • Irradiation before injection • Tumorigenicity • Safety concerns	(123–125) [NCT05734898] [NCT04623944]

ADCC, Antibody-Dependent Cellular Cytotoxicity; CAR, Chimeric Antigen Receptor; CD, Clusters of differentiation; CR, Complete Remission; DLTs, Dose Limiting Toxicities; HLA, Human Leukocyte Antigens; IL, Interleukin; LSC, Leukemia stem cells; MRD, Minimal residual disease; NK, Natural killer; NOD, Nonobese diabetic; PB, Peripheral Blood; PDX, Patient-Derived Xenograft; R/R, relapsed/refractory; SCID, Severe Combined Immunodeficiency; TCR, T-cell receptor.

### 2.2 TCR-T cell therapy

TCR-T therapy employs T-cell receptors (TCRs) from naive T cells for specificity, as opposed to relying on antibody-based CARs. These T cells are a source of TCRs that target tumor cells and subsequently modified to enhance their expression and functionality (126). The initial step in generating TCR-modified T cells involves isolating TCRs that exhibit precise recognition of leukemia-specific antigen (LSA) or leukemia-associated antigen (LAA) epitopes (127). These TCRs can be identified in T-cell clones that effectively target leukemia cells, sourced from patients’ BM or blood, or derived from healthy donor T cells stimulated with LSA or LAA peptides that restrict major histocompatibility complex (MHC) class I/II (127). Wilms’ Tumor Gene (WT1) is consistently expressed in myeloid leukemia cells, including those affected by myelodysplastic syndrome (MDS), AML, and chronic myeloid leukemia (CML). Notably, cytotoxic T lymphocytes (CTLs) specific to WT1 have been recognized in the blood of leukemia patients. Consequently, WT1 emerges as a desirable objective for CTL stimulation in leukemia immunotherapy (127–129).

The UMIN000011519 trial provided preliminary evidence that WT1-specific TCR-transduced autologous T cells are effective for refractory AML or high-risk MDS in HLA-A*24:02 patients (109, 130). Two of the eight participants exhibited decreased embryonic cells in their BM, suggesting a reversal in leukemia progression. Notably, WT1-specific TCR-T cells persisted in five patients, four surviving beyond 12 months. No toxicity-related adverse effects in healthy tissues were noticed in any participants (130). Numerous tumor-associated antigens (TAAs) and possible objectives have been recognized in preclinical TCR-T treatment investigations for AML (131, 132). Recently, additional leukemia-specific TCRs have been discovered, which bind to Formin-like protein 1 (FMNL1) and are restricted to MHC class I/II (133). Furthermore, cancer-testis antigens (CTAs), minor histocompatibility A (HA)-1, telomerase reverse transcriptase (TERT), and surviving have been recognized as TAAs and are under preclinical investigation (134, 135). HMMR/Rhamm-TCRs have been recognized as well in patients with ALL and AML (136), expanding the repertoire of potential targets for TCR-T therapy in leukemia.

### 2.3 NK cell therapy

In patients with AML, the NK cell role is often compromised, giving cancer cells the ability to avoid immune detection. NK cell immunotherapy represents a procedure to counteract NK cell inhibition, thereby enhancing their capability to eradicate cancer cells (137). Apart from antibody-mediated cell cytotoxicity (ADCC), NK cells utilize the release of cytolytic granules and cytokines to induce target cell destruction (138, 139). Romee et al. showed that NK cells display memory-like traits, evidenced by increased IFN-γ production after pre-activation with IL-12, IL-15, and IL-18, followed by a 1-3 week resting period before exposure to cytokines or K562 leukemia cells (140). These findings have sparked investigations into diverse cell sources to identify possible contenders for NK cell generation in adoptive cellular therapy. Among the cell sources being explored are cord blood, peripheral blood mononuclear cells (PBMCs), NK-92 cell lines, hematopoietic stem and progenitor cells, and induced pluripotent stem cells (141).

In order to increase the cytotoxicity and selectivity of NK cells, genetic modification through the incorporation of CAR constructs is employed (142). While CAR-T therapy is efficient for B-cell ALL and lymphoma, its use in AML treatment is challenging due to limitations and adverse effects like cytokine release syndrome (CRS) (34). In place of T cells that have been changed, NK cells with a short lifespan present a more cost-effective production option and exhibit fewer harmful side effects (143). The efficiency of CAR-NK cells in different types of cancer models is currently under active investigation. Although they show promise in the preclinical stage of AML treatment, their application is limited (144). Like T cells, NK cells able to be changed to carry identical CAR, enabling CAR-NK cells to target cancer cells for destruction. Regarding the source of CAR-NK cells, preclinical research revealed that primary human donor CD123-CAR-NK cells were less effective as CAR effector cells, while CD123-CAR-NK-92 cell lines demonstrated superior performance (145).

The challenge of identifying leukemia-specific markers that can be effectively targeted by CAR-NK cells arises from the shared phenotypic characteristics between AML and normal HSCs (143, 146). One potential target is the myeloid differentiation marker CD33, which is present in leukemia stem cells (LSCs), and over 85% of AML patient blasts (147, 148). In a preclinical study, the NK cell line YT was validated to target CD33+ AML cell lines via gene transfer of a humanized chimeric T cell receptor (cIgTCR) based on CD33 (149, 150). A subsequent phase I clinical trial confirmed the consideration of safety infusing irradiated CD33 CAR-NK-92 cells in three patients with relapsed or refractory AML, although nothing noteworthy therapeutic efficacy was noted (151). Additional probable targets for CAR-NK-92 cells include CD4 and CD7 antigens found on AML blasts. Preclinical and clinical research has focused on engineering CAR-NK-92 cells to target and eliminate CD4+ and CD7+ AML cells, specifically (152–154). Also, researchers achieved another source on which they could apply CAR structure to employ new cells like NK cells, NKT cells, and macrophage cells (155). For example, The expression level of CD47 is higher in AML stem cells and associated with suboptimal prognosis in adult AML patients (69). Recent advancements in synthetic biology and the expanding comprehension of the CD47/SIRPα axis may offer new possibilities for using engineered macrophages in clinical settings. This axis is a primary pathway that inhibits macrophage phagocytosis and activation. Therefore, CD47-CAR-macrophages hinder the CD47/SIRPα axis and also self-activate to launch an assault the CD47-positive cancer cells (156).

## 3 Challenges in immune cell therapy for AML

**Figure 1:**
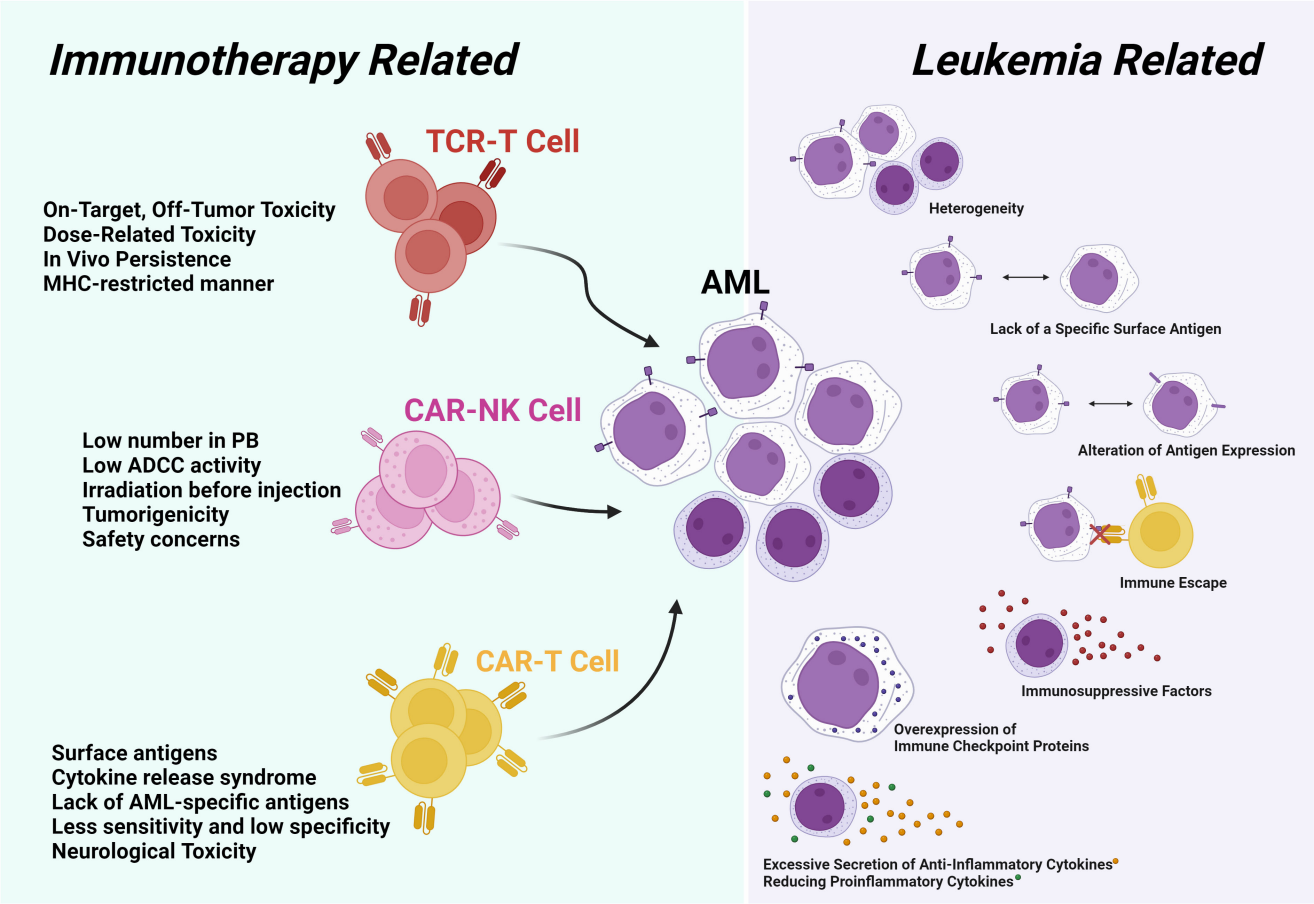
The challenges in AML can be categorized into two main groups. The first group pertains to leukemia-related challenges, encompassing issues of heterogeneity. The second group is associated with challenges related to immunotherapy methods, which may include on-target/off-tumor effects in the type of treatment method, as well as HLA matching in TCR-T cell therapy.

Immunotherapy holds immense potential in the management of AML, yet several challenges must be addressed to optimize its clinical efficacy (157). These challenges can be generally categorized into two groups: disease-specific and treatment-related. Disease-specific challenges include the heterogeneity of antigens in AML, the absence of appropriate antigens, and the BM microenvironment, which is often influenced by the existence of AML blasts (158). On the other hand, treatment-related challenges primarily revolve around ethical concerns, the potential development of cytokine release syndrome, and the continuance of cells post-injection (159) (**Figure 1**).

### 3.1 Leukemia-related challenges

AML offers a substantial challenge due to its tumor heterogeneity, primarily linked to the existence of LSCs. These LSCs perpetuate the disease through self-renewal, quiescence, and treatment-resistance mechanisms (160). Furthermore, the absence of stem cell characteristics in differentiated cells contributes to a negative impact on the surrounding environment of the tumor, consequently influencing tumor biology (161). The dynamic nature of antigen expression, which may diminish or cease during treatment, can lead to treatment insensitivity. AML’s heterogeneity arises from unique chromosomal abnormalities, gene mutations, or gene fusions, further complicating its management and therapy (162).

The challenge in treating AML is its limited efficacy and the lack of distinct surface antigens, crucial for protecting healthy hematopoietic cells. The identification of an antigen target it is essential to the biology of AML and is unique to malignant cells poses a formidable challenge (163). In contrast, CAR-T cell therapy has demonstrated significant progress has been made in In effectively treating diffuse large B-cell lymphoma (DLBCL), MM, and ALL. This success is attributed to its ability to selectively target specific surface antigens (CD19, CD22, and BCMA) (164). However, the intrinsic variability and heterogeneity of tumors present a significant hurdle in predicting patient responses, resulting in a high recurrence rate of 75%. Relapse and treatment resistance, occurring in 10-40% of cases, remain the primary complications post-treatment, thereby emphasizing the urgent need for innovative therapies (165).

Furthermore, the AML bone marrow microenvironment features various cells that suppress T-cell activity, including macrophages, regulatory T-cells (Treg), myeloid-derived suppressor cells (MDSC), and dendritic cells (DC) (166). Notably, Treg cells exhibit abnormally high expression of CD39, and increased CD73 levels have been linked to an unfavorable prognosis. Moreover, MDSC levels in BM may function as a prognostic indicator for AML (3, 167).

The proliferation of AML cells is triggered by reduced CXCL12 expression in bone marrow stromal cells, while WNT ligands from osteoblasts enhance leukemia cell survival (168). In addition to indoleamine 2,3-dioxygenase (IDO), other immunosuppressive substances, like indoleamine 2 and reactive oxygen species (ROS), contribute to immune evasion in AML (169–171). In tumor-bearing mice, the cytotoxicity ability of CD8+ T lymphocytes was discovered to be inhibited due to elevated ROS levels in immature myeloid cells generated from these animals compared to tumor-free animals (172). Similarly, an investigation conducted on human peripheral blood and BM from AML patients indicated that monocytic AML cells activated poly-ADP-ribose polymerase-1-dependent apoptosis to kill T-cells and NK cells by secreting ROS (173). Moreover, patients with AML exhibit higher expression levels of enzymes engaged in producing immunosuppressive products such as IDO. These enzymes possess the capacity to prevent T-cell reactions by causing the high expression of Treg cells (174).

AML blasts can trigger the release of pro-inflammatory cytokines, like tumor necrosis factor-α (TNF-α), IL-1β, and IL-6, in monocytes. Myeloid or lymphoid progenitor cells release two key pro-inflammatory cytokines, including IL-15 and IFN-γ, which play a crucial function in eradicating leukemia cells (175, 176). Low serum IL-15 levels immediately following allogeneic HSCT have been associated with leukemia recurrence (177). As tumors progress, elevated levels of IL-10 display strong immunosuppressive effects, inhibiting T-cell proliferation and the generation of cytokines like IFN-γ and IL-2. In the cancer field, IL-10 has been demonstrated to possess a dual biological effect, either elevating tumor development or inhibiting it (178–180).

In the tumor microenvironment, AML cells enhance the expression of immunomodulatory factors that impede the CTLs activation. These factors include transforming growth factor-β (TGF-β), arginase II, prostaglandin E2 (PGE2), CTL-associated protein 4 (CTLA-4), lymphocyte activation gene 3 (LAG3), and TIM3 on T-cells (35). As an instructional method to avoid immune surveillance systems, AML cells may induce T-cell exhaustion (181). AML relapse can occur when CD8+ and CD4+ T cells express higher levels of programmed cell death protein 1 (PD-1) following allogeneic HSCT, leading to T-cell exhaustion (182). Notably, merging T-cell therapy with drugs targeting the PD-1 immune cell has shown impressive effectiveness in treating leukemia, resulting in enhanced cytolytic activities, the memory of CD8+ T cells, and IFN-γ production (183). Therefore, inhibitory receptor-blocking strategies could be valuable treatment approaches for leukemia, as they augment the immune system’s collaborative ability (109).

Downregulation of activation ligands in addition to high expression of inhibitory receptors for NK on AML cell surface can result in cytotoxic dysregulation of NK cells. NK cells’ antileukemic replies may also be impacted through various AML receptor-ligand interactions or immunosuppressive factors secretion (184). A mouse model revealed that melding NK cells with exogenous IL-15 could boost immune effector cells to eliminate leukemia following allogeneic HSCT (137, 185). In the NCT01885897 phase I trial, leukemia patients relapsing after allogeneic HSCT showed improved CD8+ T cell and NK cell capabilities with ALT-803 (186). Furthermore, leukemia cells capable of avoid the immune system by decreasing levels of IL-1β and granulocyte colony-stimulating factor (G-CSF), which are inflammatory growth factors (187).

Despite significant expansion of infused cells *in vivo*, substantial therapeutic results are often impeded by inhibitory effects of self-HLA ligands in certain tumors, especially AML (188). Moreover, because autologous NK cells are obtained from heavily pre-treated patients, their growth and operational capabilities may not be as high as expected (189). There is a strong rationale for investigating the potential of NK cells as agents to combat leukemia. Different approaches are under development to get over the current limitations of autologous NK cell lineages. Lirilumab, an anti-KIR antibody, is meant to suppress signals causing inhibition by preventing interactions with MHC class I ligands, resulting in an upregulation of NK cells’ killing capacity (190). Additionally, the application of combination therapy, involving autologous NK cells and various anti-tumor agents, has been demonstrated to augment therapeutic responses against tumors (184). This rationale is supported by evidence from *in vitro* experiments, animal models, and clinical trials.

### 3.2 Immunotherapy-related challenges

Immunotherapy presents several challenges and benefits across different modalities, including CAR-T cell therapy, TCR-T cell therapy, NK cell therapy, and other immunotherapies. CAR-T cell therapy can lead to severe side effects like CRS and neurotoxicity, and it has limited efficacy in solid tumors because of antigen escape (191). TCR-T cell therapy confronts problems like the need for suitable TCRs and the risk of autoimmunity, alongside complex manufacturing processes (192). NK cell therapy, while capable of rapid responses, often suffers from a short-lived effect and inhibition by the tumor microenvironment (193). Other immunotherapies, like checkpoint inhibitors, can trigger immune-related unfavorable incidents and encounter resistance mechanisms due to tumor heterogeneity (194). Despite these challenges, these therapies also offer significant benefits. CAR-T cells provide a targeted approach with the potential for durable remissions, while TCR-T cells can recognize a broader range of antigens and be personalized for individual patients. NK cells benefit from a quick innate immune response and a lower risk of graft-versus-host disease (191). Additionally, other immunotherapies can enhance the immune response and be effectively combined with traditional treatments, showcasing their potential in revolutionizing cancer therapy (195). In this part, we discuss the challenges of cell-based immunotherapy.

#### 3.2.1 CAR-T cells challenges

Immunotherapeutic approaches face challenges like high “on-target off-tumor” toxicity, potentially fatal CRS, and neurological issues that hinder their effective use (146, 196). CAR-T cells can attach to substances on the cell surface without requiring antigen processing or HLA expression. A critical aspect of the production process involves choosing the right option surface antigen to target (197). The perfect target would have little to no expression on healthy tissues in order to avoid toxicity and high expression on tumor cells, exceeding CAR-T cell activation thresholds (198). Despite extensive research on the immunopathology of AML, a specific AML target remains difficult to achieve (199, 200). However, the significant danger of on-target/off-tumor activity needs to be taken care of. Prolonged myelosuppression is a concern, as most AML blast surface antigens are co-expressed by mature myeloid cells, HSCs, and other relevant tissues (200, 201). Some phase I trials have reported severe toxicities and fatalities. For instance, patients receiving CAR-T cells targeting CD33, an antigen present in most leukemic blasts and normal myeloid lineages, experienced severe pancytopenia and CRS. Additionally, excellent preclinical outcomes of CD44V6 CAR-T were accompanied by monocytopenia, likely because of the common expression of CD44v6 in circulating monocytes (202, 203). Secondary T cell lymphoma that develops after CAR-T cell therapy is rare but a noteworthy issue. The rising incidence of secondary primary malignancies, particularly myeloid neoplasms, after CAR-T cell therapy requires attention, as several reports indicate the emergence of SMNs, including MDS and AML, following this treatment. These observations suggest that prior chemotherapy and the immunosuppressive environment may elevate this risk (204, 205). The proportion of SMN after CD19 CAR-T cell therapy varies significantly, with rates reported ranging from 0.9% to 12.9%. These differences may have resulted from complex and multifactorial etiologies (205). Therefore, secondary malignancies pose a significant problem, and further research of the mechanisms participant and methods of minimizing these risks is imperative. CAR-T cell therapy has become a ground-breaking medical intervention for various diseases, but the development of secondary T-cell lymphomas and other cancers cannot be ignored.

#### 3.2.2 TCR-T cells challenges

Nevertheless, some TCR-T cell immunotherapies are currently employed to handle AML, these treatments face specific challenges (109). Until a thorough assessment of on-target/off-tumor toxicity, toxicity associated to dose, *in vivo* durability of TCR-T cells, and potential immune evasion by AML post-TCR-T injection is completed, TCR-T cell therapy usage will stay restricted (192). TCR-T cells eliminate leukemia cells by participating in their engineered TCRs with antigens presented by HLA molecules on the surfaces of these cells. Therefore, identifying neoantigens and matching HLA between donor and patient is a significant obstacle (134). One obstacle in this approach is the restriction of TCR-T cells to HLA, which is often downregulated in AML recurrence (108). However, cytokines like IFN-α, IFN-β, and IFN-γ are essential for enhancing MHC-I expression, and inserting IFN-γ into the C-domain of a TCR could circumvent MHC molecule down-regulation (206). Animal models have demonstrated that modifying TCR-T cells with pro-inflammatory cytokines like IL-15, IL-18, or IL-12 enhances persistence and exhibits a favorable safety profile when used against tumors (207, 208).

The potential of on-target and off-tumor toxicity in treatments employing adoptional swap of antigen-specific TCR-T cells raises concerns. Adoptional swap of autologous TCR-T cells has been associated with neurotoxicity and cardiac toxicity as off-target toxicity side effects in two clinical investigations (209, 210). Another challenge in TCR-T cell therapy is the restricted T cell capacity to strive and proliferate *in vivo* over extended periods, which reduces therapeutic efficacy (211). To improve T-cell persistence *in vivo*, several strategies, such as genetic modification of T-cell signaling and cytokine or pharmacological provocation of T cells, can be employed (212). An effective method for enhancing the growth and longevity of TCR-T cells is to incorporate the intracellular domain (ICD) of signaling components (such as CD28 or 4-1BB) onto CD3Ύ, rather than altering the TCR affinity (213, 214). These engineered TCR-T cells have shown improved effectiveness, extended *in vivo* lifespans, and improved proliferation (215, 216). Studies suggest that administering antigen-specific T-cells with cytokines enhances T-cell persistence and induces T-memory stem cell (TSCM) generation. Consequently, low-dose decitabine treatment of TCR-T cells may also augment phenotypic indicators of TSCM (217).

#### 3.2.3 NK cells challenges

In NK cell-based therapy, both autologous and allogeneic sources face challenges connected to prompt ex vivo expansion, low clinical-grade activation, and an absence of *in vivo* persistence (218). Combining many cytokines can play a crucial role in the activation (IL-18 and IL-21), proliferation (IL-2 and IL-15), and effector function (IFN-γ and TNF-α) of NK cells (219). The IL-15 super agonist complex, ALT-803, has proved to be a safe agent in the first in-human phase I study, emphasizing senior AML patients who relapsed following HSCT (220). In a research aimed at pre-activating NK cells with IL-2, significant *in vitro* cytolytic activity and *in vivo* persistence were observed, but no notable clinical reactions were seen. However, persistent NK cell-mediated ADCC without *in vitro* cytokine reactivation indicates that combining monoclonal antibodies with autologous adoptive NK cell transfer warrants further assessment and investigation (189, 221). Safety concerns arise from the necessity to irradiate products derived from immortalized NK lymphoma cell lines, which are utilized to cultivate NK cells, before infusion (222). Moreover, even with ADCC, the killing capacity of NK-92 cell lines may be constrained due to the absence of CD16 (FCI) and other activating killer cell immunoglobulin-like receptors (KIRs) (223). Induced pluripotent stem cells are optimal for acquiring NK cells, provided their rapid proliferation. However, they also exhibit lower CD16 levels, which might impair their ability to eliminate cancer cells. This challenge could be addressed through genetic engineering (224). Recently, innovative NK cell-based immunotherapies, like adoptive transfer and CAR-NKs, have been assessed in AML clinical trials (225). The challenges associated with NK cell therapy are depicted in **Figure 1**.

## 4 Clinical trial targets of cell-based immunotherapy for AML

AML treatment encompasses conventional chemotherapy, targeted medications, HSCT, and immune-based cell therapies. Each approach has its benefits and drawbacks. Conventional chemotherapy protocols for AML have been well-established and serve as the primary treatment for recently diagnosed cases. However, these agents can cause organ damage and hematopoietic system suppression, especially in elder patients. Emerging targeted drugs such as gilteritinib (FLT3 inhibitor) (226), enasidenib (IDH2 inhibitor) (227), ivosidenib (IDH1 inhibitor) (228), and venetoclax (BCL-2 inhibitor) (229) have demonstrated promising outcomes, but their cost may limit access for some patients. HSCT remains a feasible choice, especially for young patients with suitable donors, but carries risks of severe complications like graft-versus-host disease (GVHD) and infections (230). To develop effective targeted immunotherapies, finding an appropriate target antigen is essential. Cheever et al. listed the characteristics of a perfect antigen for targeting including immunogenicity, clinical effects, and an important part in the differentiation and proliferation of malignant cells. Its expression ought to be limited to cancerous cells; all cancerous cells, including cancerous stem cells, should express it (231). A significant ratio of patients should exhibit positive antigen tests, and malignant cells should have the antigen on their surface, consisting of several antigenic epitopes (232). In **Table 1**, we discussed the clinical studies of cell-based immunotherapy.

## 5 New potential targets of AML for cell-based immunotherapy

**Figure 2:**
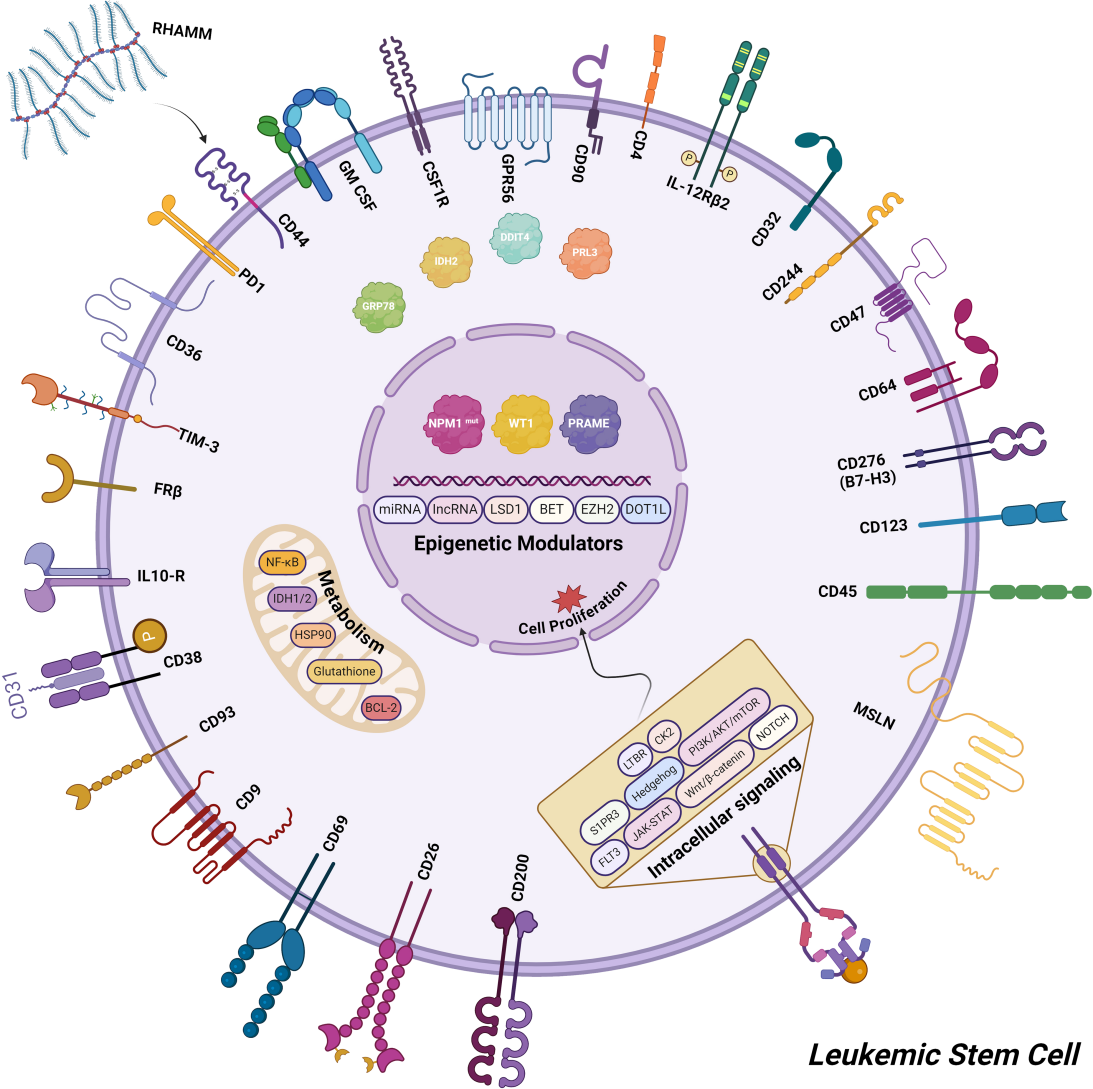
The updated targets for AML cell-based immunotherapy encompass a range of potential candidates, including cell surface antigens, proteins involved in various signaling pathways, key factors in cell metabolism, and epigenetic modulators.

Based on the challenges mentioned above, various solutions were considered. One crucial solution involves identifying a new target capable of specifically and accurately attacking cancer cells, while also preventing the protection of the stromal cells in the BM niche. The progression of treatments for AML has been hindered by the diversity and high frequency of disease relapse, emphasizing the critical demand for novel therapeutic options. Researchers are investigating diverse strategies for managing AML, and while some are more promising than others, each can cause valuable treatments (233) (**Figure 2**).

**Figure 3:**
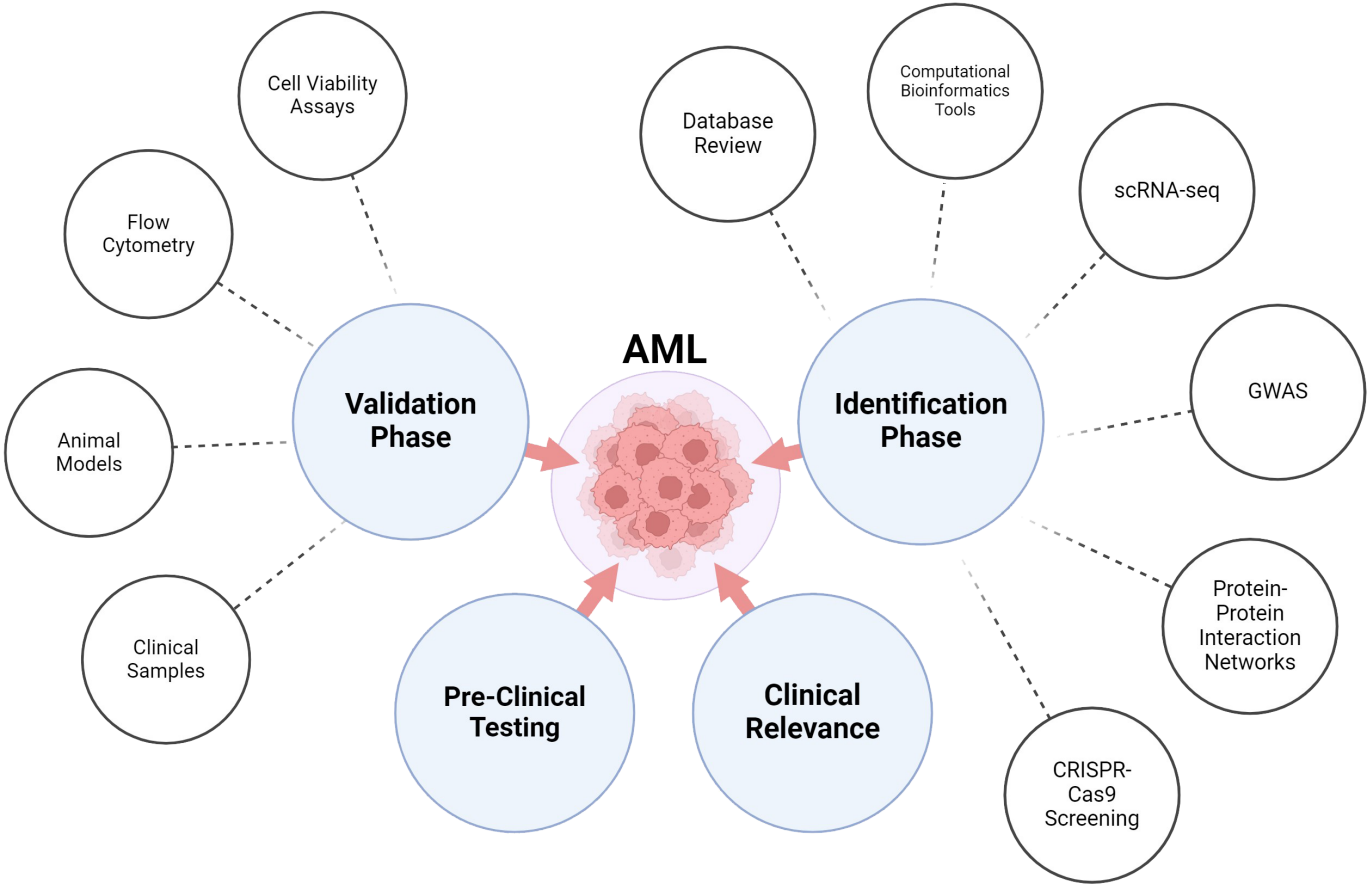
Find the potential targets for AML immunotherapies.

Computational models have recently emerged as useful instruments for the *in silico* and systematic analysis of significant biological mechanisms and patient remarks in cancer immunotherapy. These models are according to empirical justifications and mathematical simulations with clinical data input (234). However, computational modeling of CAR-T cell therapy remains in its early phases, and there are limited applications for model-informed response prediction. For instance, utilizing information from xenograft mouse models, a multiscale pharmacokinetic-pharmacodynamic model based on physiological principles was created to quantitatively investigate the connection between CAR affinity, antigen abundance, tumor cell depletion, and CAR-T cell expansion (235, 236). Other approaches model factors influencing CAR-T cell dynamics, such as ecological dynamics regulating expansion and exhaustion, signaling variability in cell states, lymphodepletion effects on expansion, and competition between CAR-T and normal T-cells (237, 238). Recently, Liu et al. created a model retrospectively to describe clinical kinetics of CAR-T cells in relation to reaction status, patient populations, and tumor types (236). However, computational models often lack comprehensive analysis of clinical trial data, and a predictive model based on clinical data to forecasts of patient responses to CAR-T cell treatment are mostly lacking (239) (**Figure 3**).

The significance of the optimized interaction among AML blasts, the hematopoietic niche, and immune cells has been demonstrated to play a critical function in AML expansion and progression in recent years (240). The mechanisms behind the capacity of AML cells to evade the immune system and induce systemic tolerance have been better understood (241). These tolerogens provide an immunosuppressive microenvironment that impairs anti-leukemia immune responses and decreases the effectiveness of both traditional and novel treatments (242). Clinically applicable novel medications, examples include immune checkpoint and macrophage checkpoint inhibitors, have emerged to target these pathways and enhance anti-tumor immunity (243). In the present clinical trials primarily focus on lineage-restricted antigens, but newer approaches, such as split and dual targeting, aim to target leukemia-specific intracellular antigens (244). Bispecific antibodies and adapter CAR-T cells offer temporary exposure, improved safety, and multitargeting capabilities against antigen-escape variants. Bispecific antibodies encompass several types, such as bispecific T-cell engagers (BiTEs), bispecific killer-cell engagers (BiKEs), dual-affinity retargeting antibodies (DARTs), and tandem diabodies (TandAbs). These antibodies feature two antigen recognition sites that help redirect tumor cells toward immune cells (245). To overcome the AML’s molecular variability and the inherent variety of AML blasts, a combined or sequential approach involving immunotherapy, chemotherapy, and molecular therapy is likely to be the most effective (246). This three-pronged strategy aims to control the disease and prevent relapse. To improve AML cure rates, it is also essential to develop new methods for tracking MRD and identifying potential recurring clones at an early stage, in addition to early response biomarkers and genomic profiling (247). Extending the utilization of CAR-T cells to target intracellular antigens could benefit a broader range of cancer patients. Despite this, clinical trials for immunotherapy and chemotherapy combinations are yet in their early years, these treatments hold promise for AML patients. Through complement-dependent cytotoxicity, innate immune system activation, and ADCC, immune agents cause AML cell death (248, 249).

In the last ten years, single-cell technologies have undergone a dramatic evolution, producing a wealth of single-cell expression data that precisely delineates the transcriptomic topography of both cancerous and healthy cells (250). This rich data reservoir, largely unexplored, is capable of creating new treatments, especially in the background of CAR-T cell development and novel antigen predictions (251). These advancements facilitate precise on- and off-tumor antigen predictions, providing unparalleled resolution and distinct perspectives into both malignant and healthy cells. For example, the AML antigens CD33 and CD123 did not meet our stringent overexpression standards for malignant hematopoietic stem and progenitor cells (HSPCs), most likely because of their expression in healthy HSPCs as well (80, 252). Furthermore, endothelial and other lung cell types exhibited high CD123 expression levels, potentially leading to on-target off-tumor toxicity. An investigation was conducted using a group of 15 AML patients to find potential antigens for CAR-T cell treatment. In order to accomplish this, a technique called single-cell RNA sequencing (scRNA-seq) (198, 252). A comprehensive transcriptome atlas was created by utilizing publicly accessible datasets. This atlas includes more than 28,000 malignant and healthy BM cells taken from patients, as well as over 500,000 healthy cells from nine important human tissues (252). The atlas underwent screening to identify cell surface antigens that are expressed on cancerous cells but have low expression on healthy cells, specifically T lymphocytes (253). Through the use of stringent criteria, researchers have successfully identified two CAR-T cell targets in AML that were previously unknown: CD86 and the colony-stimulating factor 1 receptor (CSF1R) (254, 255). CAR-T cells were produced against both targets and assessed for effectiveness using primary AML blasts and other patient-derived models, both *in vitro* and *in vivo* (252). For *in vitro* safety studies, we used advanced primary cell cultures of cell types that express the target. These cultures showed a higher ability to distinguish between different cell types compared to well-known anti-CD33 CAR-T cells (252). To address concerns regarding safety, various *in vivo* models were employed. These results establish the foundation for the clinical advancement of the CAR candidates and emphasize the potential for practical application of an objective scRNA-seq-based screening technology (252).

Recent innovations in immune-based therapies for AML concentrate on utilizing the immune system to tackle the disease. These approaches focus on targeting intrinsic and surface antigens of cancer cells, additionally modifying the leukemic microenvironment to reduce immune evasion like HLA loss and T-cell exhaustion during cancer progression (256). For example, in study on dual CAR-T, a new combination platform of twofold aiming by an antibody-T cell receptor (AbTCR) and a chimeric costimulatory signaling receptor (CSR) to two different antigens, wherein the cancer cells express both antigens simultaneously, but not together on normal cells. In this study two different antigens Wilm’s tumor 1 protein (WT1) and CD33 were targeted that both are highly expressed on most AML cells. These data suggest that this amalgamation of a AbTCR CAR and CSR might work well as a tactic to lessen toxicity and enhance specificity and clinical results in adoptive T cell therapy in AML (257). So, the search for novel targets and therapies for AML underscores the necessity for innovative strategies. Integrating single-cell technologies and computational modeling presents promising pathways for identifying effective CAR-T cell targets. A multi-faceted approach combining immunotherapy, chemotherapy, and advanced tracking methods is crucial for improving treatment efficacy and minimizing relapse rates.

Advancements in genomics and precision medicine have profoundly shaped the strategic selection of targets for immune therapies, revealing intricate genetic and molecular foundations of diseases, notably cancer. This knowledge permits the precise identification of particular genetic aberrations and molecular routes susceptible to immune therapies, increasing their effectiveness while reducing off-target effects. The employment of high-throughput sequencing technologies enables comprehensive analysis of tumor genomes, which identifies tumor-specific modifications that give rise to neoantigens. These neoantigens serve as optimal targets for immune interventions because of their tumor specificity and recognition by the immune system as foreign entities (258). Furthermore, computational tools leveraging genomic data proficiently predict potential neoantigenic mutations based on the affinity of altered peptides for major histocompatibility complex (MHC) molecules, essential for antigen presentation to T cells (259). A notable correlation can be observed between high tumor mutational burden (TMB) and the proliferation of neoantigens, positioning tumors with elevated TMB as prime candidates for immune checkpoint inhibitors. Genomic sequencing quantifies TMB, thus directing the deployment of these inhibitors. For example, elevated TMB is linked with enhanced responses to checkpoint inhibitors since mutation-rich tumors are more likely to generate recognizable neoantigens (260). Additionally, microsatellite instability (MSI) suggests a heightened mutational load and an abundance of neoantigens, rendering such tumors suitable for immune therapy (261). Genomic assessments can pinpoint mutations essential for the survival of cancer cells (oncogenic drivers). Attacking these drivers with immune therapies through direct targeting or blocking reliant pathways has proven efficacious. For example, although direct drug targeting of KRAS gene mutations has been challenging, immune therapies tailored to attack KRAS-mutant cells are under development. Integrating genomics into precision medicine also aids in unearthing novel immune targets by analyzing the genetic landscape of tumors (262). Identifying mutations in pathways such as the JAK/STAT pathway has catalyzed the development of therapies that enhance immune checkpoint blockade efficacy by thwarting immune evasion mechanisms (263). Advances in transcriptomics have further enabled the exploration of gene expression in the tumor microenvironment, revealing the presence and activity of immune cells such as T cells, macrophages, and regulatory T cells. This information will help with the decision-making of appropriate immune therapies, including checkpoint inhibitors or adoptive cell therapies (227, 264). Single-cell sequencing technology, which analyzes gene expression at the cellular level within the tumor microenvironment, assists in pinpointing specific cell populations that may promote or inhibit an immune response, thereby guiding immune modulator selection (265). Furthermore, genomics has facilitated the creation of biomarkers that guide the selection and fine-tuning of immune therapies. Biomarkers such as MSI and specific gene expression profiles tailor immune therapies to individual patients, ensuring that treatments are optimally matched to their unique genetic profiles (266). Precision medicine employs genomic insights to categorize patients by the molecular attributes of their tumors-this stratification aids in selecting optimal immune therapies that enhance effectiveness and diminish side effects (267). Personalized cancer vaccines, which are designed to target unique neoantigens within a patient’s tumor, provoke specific immune reactions. However, some tumors have evolved resistance to these therapies. Genomic analysis exposes resistance mechanisms, such as mutations that disrupt antigen presentation or elevate immune-suppressive pathways. Comprehending these mechanisms fosters the development of combination therapies that triumph overy such resistance. The integration of genomic information with immune profiling enables the development of multifaceted therapies. These therapies address various elements of tumor biology, thereby improving clinical outcomes and lessening unwanted effects (268, 269). However, despite these advances, significant challenges remain in applying genomics and precision medicine in selecting immune therapy targets (266). The inherent heterogeneity of tumors and the dynamic interactions within the immune system and tumor microenvironment often hinder the consistent identification of stable, effective targets (270).

## 6 Conclusion

In conclusion, this work highlights the promising potential of various immunotherapeutic strategies in targeting AML while emphasizing the need for a more thorough comprehension of the tumor microenvironment and its interactions with immune cells. In the past three to five years, significant advancements in AML immunology, coupled with technological breakthroughs, have led to innovative therapeutic strategies for AML-targeted T cells. Despite the plethora of continuing investigations, T-cell immunotherapies for myeloid malignancies continue to be available in their nascent stages, poised to evolve and refine AML immunotherapeutic in the coming years. A targeted investigation of biomarkers at various phases-pre-therapy, on-therapy, and relapse—will accelerate clinical advancements, improve immune toxicity management, validate new checkpoints and AML-specific targets, elucidate mechanisms of immune resistance, and identify likely responders. Identifying and implementing these treatments in optimal clinical settings, such as MRD and low-mortality illness, will be essential. Innovative techniques like mass cytometry, single-cell RNA and DNA sequencing, and single-cell cytokine analysis will provide critical insights into non-T-cell compartments in immune responses and tumor microenvironments, guiding sequential or combinatorial immune therapy strategies. Overall, this comprehensive understanding of immunotherapy’s role in AML, alongside biomarker-guided strategies, positions us for an exciting and potentially fruitful decade ahead for AML immunotherapies.

## Glossary

ADCC: Antibody-dependent cell-mediated cytotoxicity
ALL: Acute lymphoblastic leukemia
AML: Acute myeloid leukemia
BCL-2: B-cell Lymphoma 2
BCMA: B-cell maturation antigen
CAR: Chimeric antigen receptor
cCAR: Compound CAR
CD: Cluster of differentiation
CLEC12A: C-type lectin domain family 12 member A
CLL1: C-type lectin-like molecule-1
CML: Chronic myeloid leukemia
CRS: Cytokine release syndrome
CSF1R: Colony-Stimulating Factor 1 Receptor
CTAs: Cancer-testis antigens
CTL: Cytotoxic T lymphocyte
CTLA-4: Cytotoxic T-lymphocyte-associated protein 4
CXCL12: C-X-C motif chemokine ligand 12
DC: Dendritic cells
DLBCL: Diffuse large B-cell lymphoma
FLT3: Fms-like tyrosine kinase 3
FMNL1: Formin-like protein 1
G-CSF: Granulocyte colony-stimulating factor
GVHD: Graft-versus-Host Disease
HA-1: Minor histocompatibility antigen A-1
HLA: Human leukocyte antigen
HMMR/Rhamm: Hyaluronan-mediated motility receptor
HSC: Hematopoietic stem cell
HSCT: Hematopoietic stem cell transplantation
IDH: Isocitrate Dehydrogenase
IDO: Indoleamine 2,3-dioxygenase
IFN: Interferon
IFN-γ: Interferon-gamma
IL: Interleukin
KIRs: Killer cell immunoglobulin-like receptors
LAA: Leukemia-associated antigen
LAG-3: Lymphocyte-activation gene 3
LILRB4: Leukocyte immunoglobulin-like receptor B4
LSA: Leukemia-specific antigen
LSC: Leukemia stem cell
MDS: Myelodysplastic syndrome
MDSC: Myeloid-derived suppressor cells
MHC: Major histocompatibility complex
MM: Multiple myeloma
MRD: Minimal Residual Disease
NK: Natural Killer
NKG2D: Natural killer group 2, member D
PBMC: Peripheral blood mononuclear cell
PD-1: Programmed cell death protein 1
PD-L1: Programmed death-ligand 1
PGE2: Prostaglandin E2
ROS: Reactive oxygen species
Siglec-6: Sialic acid-binding Ig-like lectin 6
TAA: Tumor-associated antigen
TCR: T-cell receptor
TERT: Telomerase reverse transcriptase
TGF-β: Transforming growth factor-beta
TIM3: T cell immunoglobulin and mucin-domain containing 3
TNF-α: Tumor necrosis factor-alpha
Treg: Regulatory T-cells
TSCM: T-memory stem cells
WT1: Wilms’ Tumor
